# A Systematic Review of Soil Properties to Support Mycotoxin Model Development with In-Field Soil Sensing

**DOI:** 10.3390/s26134044

**Published:** 2026-06-25

**Authors:** Eleonora Granata, Marco Camardo Leggieri, Daniele Trinchero, Paola Battilani

**Affiliations:** 1 Dipartimento di Scienze delle Produzioni Vegetali Sostenibili, Università Cattolica del Sacro Cuore, Via Emilia Parmense 84, 29122 Piacenza, Italy; 2 Plant Health Modelling Research Center—PHEM, Università Cattolica del Sacro Cuore, Via Emilia Parmense 84, 29122 Piacenza, Italy; 3 Dipartimento di Elettronica e Telecomunicazioni, Politecnico di Torino, Corso Castelfidardo 39, 10129 Torino, Italy

**Keywords:** fungal community, aspergillus, fusarium, crop, water content, pH, temperature, electrical conductivity, smart agriculture, IOT

## Abstract

Recently, mycotoxin prediction has mainly relied on meteorological data and crop physiology. The contribution of soil characteristics as additional environmental variables remains largely unexplored. A systematic literature search was carried out to analyze the latest research (from 2020 to 2025) on the relationship between soil properties (temperature, water content, pH, and electrical conductivity), fungal communities (particularly *Aspergillus* and *Fusarium*), and different crops (mainly peanut, wheat, and maize). Measurement methodologies were analyzed, with a focus on the use of in-field soil sensors in correlation studies and predictive models. Disease incidence and mycotoxin occurrence were related to stressful soil conditions, such as different pH levels, wetness or drought, and temperatures above 25 °C. Other external variables (crop and field management) must also be considered. Laboratory equipment was primarily used in correlation studies, with limited in-field sensor implementation. Although recent predictive models included soil properties as effective inputs, they mostly relied on satellite data. However, real-time conditions and fluctuations, which can be captured by in-field soil sensors, are essential for training new functional models. To monitor soil properties, IoT technologies must be considered, but their implementation is still not sufficient to collect widespread data. Therefore, groundwork is needed to fill this gap with high-quality soil data for future in-field experimentation.

## 1. Introduction

Mycotoxins are toxic secondary metabolites produced by several fungi. Mycotoxigenic fungi can infect crops both pre- and post-harvest, and they are commonly associated with commodities such as cereals, nuts, dried fruits, and spices. Susceptible crops range from wheat and maize to peanuts and walnuts, but they may also include fruits and legumes, such as apples, peaches, or soybeans. Contamination can lead to products exceeding international safety limits, resulting in significant economic losses along the food and feed supply chains, as well as posing health risks to humans and animals [1].

The most widely studied and regulated mycotoxins include *Aspergillus* toxins, such as aflatoxins (AFs) and ochratoxins (OTs), and *Fusarium* toxins, such as fumonisins (FBs), deoxynivalenol (DON), and zearalenone (ZEN). AFs are strongly carcinogenic metabolites produced by *Aspergillus* species within section *Flavi* [2] and represent a major global food safety concern [3]. FBs are the main contaminants of maize and maize-derived products, and they have been associated with several human and animal disorders, including neural tube defects, cancer, and pulmonary edema [4]. DON has been reported to harm the immune system of animals, change cell morphology and function, and induce oxidative stress-mediated cytotoxicity [5]. ZEN can be found in different crops grown in temperate climate regions, including corn, wheat, and barley [6]. ZEN exhibits immunotoxic, hepatotoxic, and xenogenic effects [7]. The International Agency for Research on Cancer (IARC) has designated many mycotoxins as causative agents of cancer in humans: In 2012, several AFs, including AFB1 and AFB2, were classified as class 1 carcinogens or as carcinogenic to humans; in 2002, FB1 and FB2 were classified as class 2B carcinogens or as possibly carcinogenic to humans; and in 1993, OTA was also included in class 2B. DON and ZEN are classified by the IARC in Group 3, meaning that they are not classifiable with respect to their carcinogenicity to humans [4,8].

Crop infection and contamination depend on the relationship between the fungi, the host crop, and the environment [9], the well-known disease triangle. For example, *A. flavus* and *A. parasiticus* are able to readily infect maize and peanuts under drought and high air temperature conditions (29–35 °C), with plant stress at pollination further increasing susceptibility and insect larvae enhancing fungal infection [9,10]. The relationship between air temperature and humidity was also highlighted regarding *Fusarium* contamination in small grains [11], where rainfall during anthesis is crucial for the initial infection process [11]. Wet conditions and heavy precipitation render the raindrop transfer of macroconidia to the upper parts of the plant easier [12], which also occurs within optimum temperature ranges between 10 and 25 °C [12]. For ZEN, optimal production has been associated with moderate air temperatures (20–25 °C) and high relative humidity [7]. However, when fungi are exposed to environmental stresses in the field, such as extreme air temperatures or water stress, mycotoxin production can significantly increase [3,4,7].

Several predictive models have been developed to support farmers by predicting mycotoxin risk under field conditions, using meteorological factors such as precipitation, air temperature, and humidity as inputs [13,14]. Mechanistic and AI-based predictive models were developed in Europe to forecast mycotoxin outbreaks in maize grown in Serbia and Italy [3,14], with particular attention to AF (AFLA-maize) [14,15] and FBs (FER-maize) [14,16] contamination. A predictive model based on farmers’ AF-related insurance claims was also developed in the US, along with another environmental-based model in Georgia, USA [3]. Empirical and mechanistic models have also been employed to investigate the occurrence and interaction of *Fusarium* head blight with other environmental variables and the host plant [1].

However, weather conditions are not the only environmental factors influencing the infection of mycotoxigenic fungi in plants. Soil, for instance, is considered the main reservoir for *Aspergillus* inoculum [9], even though the distribution and growth of *Aspergillus* species in soil depend on many factors, including soil type and water retention rate, crop rotation, and insect attacks [9]. Members of the *Fusarium* genus usually survive in soil or plant debris [17]. Therefore, along with the climate, soil physicochemical parameters can also determine the distribution and activity of pathogenic *Fusarium* species [17].

Given the growing recognition of soil as a key component of the disease triangle, this systematic review aims to gather and synthesize current knowledge on how soil physicochemical properties (pH, electrical conductivity or EC, moisture, and temperature) influence fungal communities—particularly mycotoxigenic fungi—and to assess how such data are collected, with specific attention to the implementation of in-field soil sensing and predictive modeling. To our knowledge, this is the first review that jointly evaluates soil physicochemical properties, their reported relationships with mycotoxigenic fungal communities, and the potential role of in-field soil sensing as a data source for mycotoxin predictive modeling.

## 2. Materials and Methods

A systematic literature review was conducted according to the Preferred Reporting Items for Systematic Reviews and Meta-Analyses [18] and registered in the Open Science Framework public registry [19]. Scopus and Web of Science (WOS) were selected as primary sources of information. The publication time range was set from 2020 to 2025. The two databases were last accessed for this study on 10 March 2026.

The search regarding soil physicochemical properties, fungal occurrence, and selected crops was based on the keyword “soil”. The keyword “electrical conductance” was also added for search completeness. The full query was as follows: (soil OR terrain) AND (physicochemical OR properties OR characteristics OR pH OR water OR “water content” OR humidity OR moisture OR temperature OR “electrical conductance” OR “electrical conductivity” OR salinity OR “sodium chloride” OR “NaCl”) AND (fungi OR Aspergillus OR Fusarium OR mycotoxins OR aflatoxins OR fumonisins OR deoxynivalenol) AND (peanut OR groundnuts OR wheat OR maize OR grain OR kernel) AND NOT (mycorrhiza OR mycorrhizal).

The literature search regarding the state of the art of field sensors utilized a different query to obtain comprehensive information from each database. All queries were centered around the word “sensing” and the main physicochemical properties retrieved previously. The keyword “electrical conductance” was also added for search completeness. The main keywords were as follows: (“soil sens*” OR “terrain sens*”) AND (pH OR temperature OR humidity OR “water content” OR moisture OR “electrical conductivity” OR “electrical conductance”).

The obtained literature was preliminarily evaluated based on the title and the abstract, followed by a full-text review, in accordance with the inclusion/exclusion criteria at each step. Admissible records were maintained for further analysis, while the rest of the data were not considered. To investigate how soil properties can influence mycotoxigenic fungi, the research included had to satisfy the following: (i) contain the term(s) “fungal communities”, “microbial communities”, “soil microorganisms”, “soil characteristics”, “soil properties”, or any keyword related to mycotoxigenic fungi in the title or the abstract; (ii) address microbial and/or fungal communities in relation to soil characteristics; (iii) contain or report original data on the effects of soil properties on fungal communities; (iv) be published in journals, proceedings, or other forms of works from competent authorities/organizations. Articles containing the following terms and/or characteristics were excluded: (i) analysis focusing on mycorrhizal communities and/or mycorrhizal presence. According to the obtained initial articles and through an ancestry approach, publications prior to the set time limit range of the search were also considered for retention when appropriate and/or notable for the final purpose of the research.

To investigate the state of the art regarding in-field soil sensors, the included articles had to satisfy the following criteria: (i) contain or refer to the term(s) “field”, “station”, “sensor”, “probe”, or “real-time” in the title or the abstract; (ii) address the use of soil sensors in the field; (iii) report how soil sensors work or have been used; and (iv) be published in journals, proceedings, or other forms of works from competent authorities/organizations. To be retained, an article also could not include the following: (i) “remote sensing”, “remote”, “satellite sensing”, “satellite”, and any other keywords related to remote sensing as the main part of the title, abstract, or full text; and (ii) “proximal sensing”, “proximal”, “in situ sensing”, “in situ”, or other keywords related to in situ proximal soil sensing as the main part of the title, abstract, or full text.

All records were single-handedly screened to limit bias according to whether they met the inclusion/exclusion criteria. All final paper citations were uploaded to Zotero. The two queries and retained data were maintained independently for further analysis. Duplicate records within the two queries were manually handled, and one duplicate was excluded at the first step, along with non-admissible data. Each study that met the inclusion and exclusion criteria for the first query was retained and analyzed qualitatively based on the reported trends and/or correlations between the soil fungal community and soil physicochemical properties. Each correlation or statistical value found in the studies was double-checked for reported statistical significance. Not statistically significant correlations were labeled as not correlated, while unmeasured correlations were reported as trends. Each study that met the inclusion/exclusion criteria for the second query was retained and analyzed qualitatively based on the reported use of soil in situ sensing technology and which reported technology was used or analyzed. Unknown measuring techniques were labeled as Not Determined. Any missing value was not considered in the final analysis.

Journal lists and authors’ keywords were retrieved from the systematic literature search metadata, according to the database output. Measurement locations and methodologies, and the studies’ final results were retrieved manually from the articles’ Materials and Methods and Results sections. Each retained study was screened for additional information and possible bias, such as soil property range of measurement, specific fungal taxonomical group of interest, crop(s) of interest, field management, and/or treatment.

The number of studies that measured one (or more) soil property(ies) in their analysis and the number of studies that used one (or more) reported measurement methodology(ies) were calculated and further analyzed as percentages. The number of retained soil sensors that measured a soil property in the analysis and the number of soil sensors that used the reported measurement technology were calculated and further analyzed as percentages. All data were analyzed using Microsoft Excel (Microsoft 365 MSO v2504) and RStudio (v2023.06.2; R v4.4.2) to better summarize measurement technologies, measured soil properties, and the soil sensors and sensing technologies used per retained article. The bar graphs, network graphs, pie charts, and the article map were created using RStudio and the R programming language, while the network graph was created with Gephi v0.10.

## 3. Results

**Figure 1:**
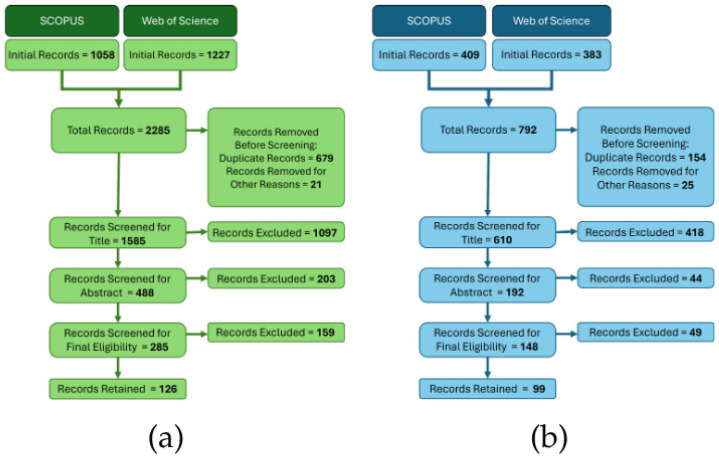
The literature search workflow with Scopus and Web of Science databases following the Preferred Reporting Items for Systematic Reviews and Meta-Analyses (PRISMA 2020, Table S3) on (**a**) soil properties and fungal presence and (**b**) in-field soil sensors’ state of the art. Duplicates and other non-admissible records were partially condensed into a single data entry or removed, respectively; then, the articles were screened according to the title, abstract, and the whole text. The final retrieved articles were considered during the drafting of this review.

**Figure 2:**
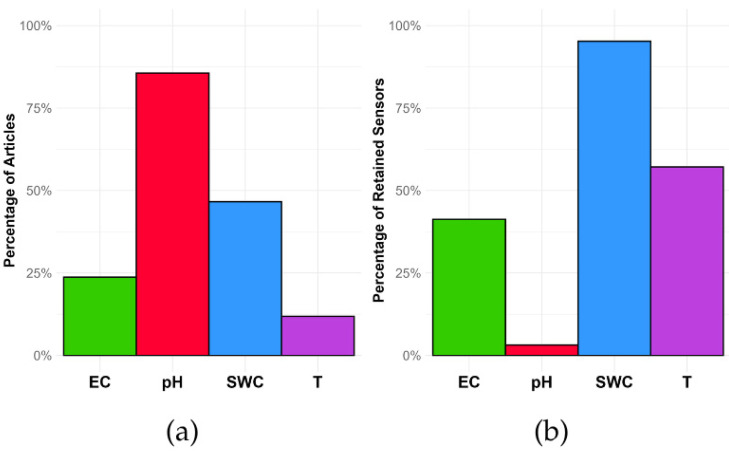
(**a**) Percentage of retrieved articles and (**b**) retrieved soil in-field sensors that measured soil electrical conductivity (EC), pH, soil water content (SWC), and soil temperature (T). The articles considered were the articles retained last within the literature search. The bar chart was created using R and RStudio.

**Figure 3:**
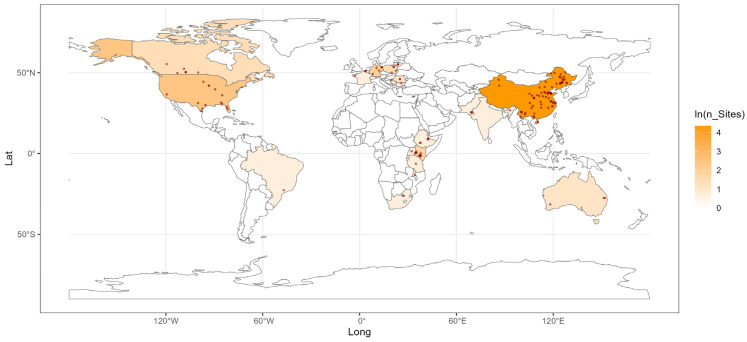
Map of retrieved article locations for soil data sampling and/or analysis, provided by the authors (red dots). Each country is colored according to the number of referring articles (logarithmic scaling). The map was created using R and RStudio.

**Figure 4:**
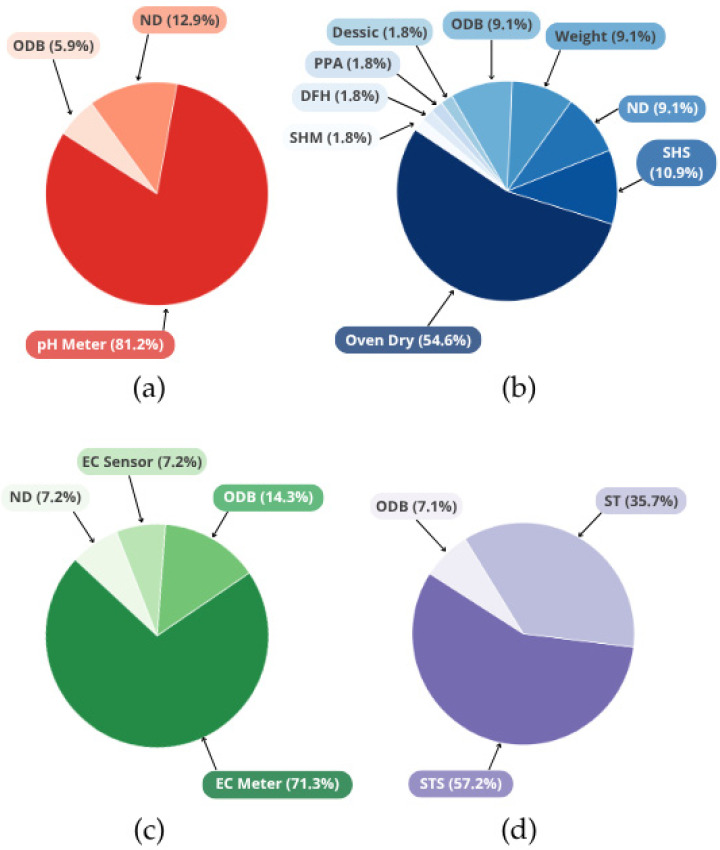
Pie charts of the methodologies used for measuring the following: (**a**) soil pH; (**b**) soil water content (SWC); (**c**) soil electrical conductivity (EC); and (**d**) soil temperature. These pie charts refer to the retained articles on the influence of soil properties on the fungal community and mycotoxigenic fungi. Abbreviations: (**a**) ND, not determined; ODB, online database; (**b**) SHS, soil humidity sensor; ND, not determined; ODB, online database; Weight, weighing; DFH, depth of first horizon; Dessic, desiccators; PPA, pressure plate apparatus; SHM, soil humidity meter; (**c**) ODB, online database; ND, not determined; (**d**) STS, soil temperature sensor; ST, soil thermometer; ODB, online database.

**Figure 5:**
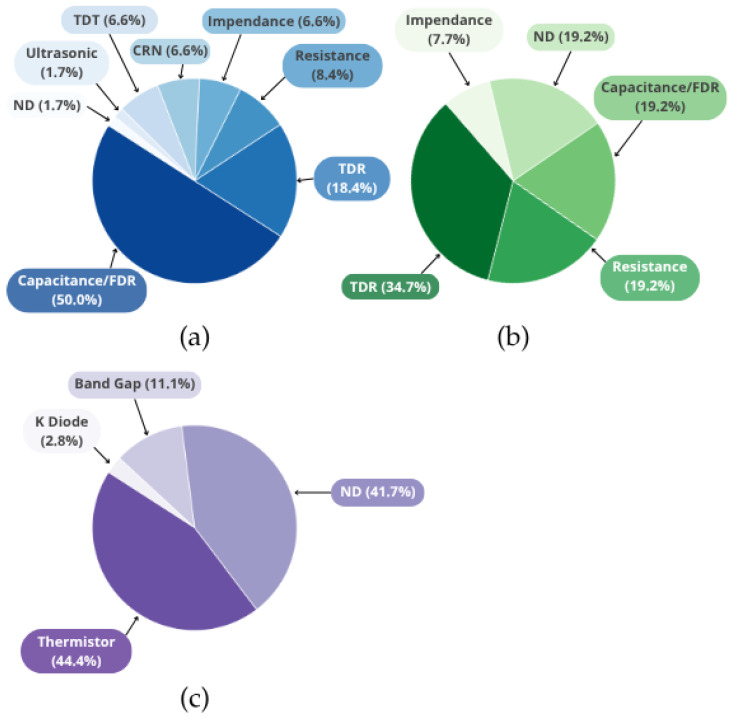
Pie chart with the percentages of soil sensing techniques utilized by the soil sensors used within the retained articles for measuring (**a**) soil moisture, (**b**) soil electrical conductivity (EC), and (**c**) soil temperature. Abbreviations: (**a**) TDR, time-domain reflectometry; FDR, frequency-domain reflectometry; CRN, cosmic ray neutron; TDT, time-domain transmission; (**b**) TDR, time-domain reflectometry; FDR, frequency-domain reflectometry; ND, not determined; (**c**) ND, not determined.

The initial searches retrieved 2285 articles on soil physicochemical properties and 792 on in-field soil sensing, for a total of 3077 initial articles. After manually removing duplicates and erratum notes, titles, abstracts, and full texts were screened following PRISMA guidelines [18]. Ultimately, 126 articles addressing soil properties and 99 addressing soil sensing were retained for analysis, for a total of 225 retained articles. The whole process of article exclusion and inclusion is shown in Figure 1. The percentage of articles and the percentage of in-field soil sensors measuring the soil properties considered are shown in Figure 2. The provided journals, authors, and keywords are also analyzed in Figures S1–S3. The soil sampling and/or field locations are mapped in Figure 3. The different methodologies used in the articles to gather soil data are also collected in Table S1 and shown in Figure 4. The different methodologies used by in-field soil sensors to acquire soil data are also collected in Table S2 and shown in Figure 5. The findings are summarized in the paragraphs below.

### 3.1. Main Physicochemical Properties Affecting Soil Fungal Populations

Retrieved articles were qualitatively examined for possible correlations between fungal presence, disease incidence, and/or in-field mycotoxin occurrence and soil characteristics, excluding studies focused on the application of in-field soil sensors to measure those physicochemical properties. Any trend that was not considered statistically significant was deemed not correlated, and each trend that was not directly calculated in the studies was evaluated as a qualitative trend. The methodologies used to measure each physicochemical property are summarized in Figure 4.

The parameters used to describe the fungal population were fungal abundance, fungal community diversity, composition or richness, and structure. Fungal abundance can be measured through phospholipid fatty acid (PLFA) characterization in the soil or through DNA extraction and high-throughput sequencing from the soil. The quantity of fungal PLFA biomarkers (expressed in nmol) or fungal DNA sequences (expressed as a raw number) per gram of dry soil was reported as fungal abundance [20,21,22,23,24,25]. If the abundance of bacterial PLFAs was also calculated, the two values can be compared through the fungi-to-bacteria ratio, a tool used to analyze the relationship between fungal and bacterial abundance in a given sample [26]. Fungal diversity can be measured to explain how heterogeneous a given community is in its components. Two important aspects of fungal diversity can be explained by fungal richness (also called composition), or the number of different species present in each sample or environment, and by fungal evenness, or the distribution of individuals between different species in a given sample or environment. The most used methods to calculate these variables are biological indices such as the Shannon–Wiener index, the Simpson index, the Chao1 index, the Pielou index, and the ACE index, all obtained through statistical software [21,27,28,29,30,31,32,33]. All these indices can explain richness (ACE, Chao1), evenness (Pielou), or the entire diversity of the community (Shannon–Wiener, Simpson). Fungal community structure is more complex and diverse than the other variables. It is used to represent the different relationships between individuals and/or groups in the same sample and how those relationships may vary with different conditions. Fungal community structure can not only be measured through principal component analysis (PCoA) and similar statistical analyses but also through network analysis [34,35,36,37,38]. A network’s topological properties (number of nodes, links, clusters, centrality, etc.) can be useful in determining statistical differences between two conditions or treatments [39,40]. All results are reported per soil physicochemical property in the paragraphs below. The findings on reported positive/negative correlations between soil properties, mycotoxigenic fungal presence, and mycotoxin contamination are summarized in Table 1.

#### 3.1.1. Soil pH

Soil pH is one of the key chemical properties shaping the microbial community [64,65,66], including fungi. Since pH is, by definition, the expression of the acidity or alkalinity of a solution, measuring the pH of the soil can provide relevant information about the state of the soil itself, from whether there are free anions and cations to whether the present microbial community can find suitable conditions to assimilate nutrients and grow [67,68]. It is important to underline how soil pH is not a static characteristic: it can fluctuate over time due to external conditions, such as weather factors (rainfall) or human practices (fertilization, irrigation, or mulching) [34,58].

Eighty-five percent of the retrieved articles measured pH while investigating how soil properties influenced the fungal community, mainly using laboratory techniques and instruments such as pH meters (Figure 2a and Figure 4a).

Several studies observed that soil pH can affect the fungal community in different ways [28,37,38,52,69,70,71,72,73,74,75,76,77]. Based on several reports from different geographic areas and crops, variations in fungal community composition [25,78], abundance [21,68,79,80,81], diversity [21,77,82,83,84,85,86], or structure [39,53,87,88,89,90] can be correlated with variations in soil pH. However, according to the reported soil pH ranges, it is difficult to state whether these correlations are mainly positive (71.4% of found correlations) or negative (28.6% of found correlations). Three studies have stated that fungal abundance is positively correlated with soil pH variations between pH 4.4 and 7 under different field management (fertigation, manure, or biochar application) for different crops (rice, maize, potato–winter rye–maize rotation) [21,79,81] while it was negatively correlated in a similar pH range (4.7–6) in peanut and maize fields [68,78]. One study also extended the positive correlation to soil pH ranges of 8–9 in a tomato greenhouse with degraded soil [91].

Fungal diversity indices can also be differently correlated with variations in soil pH within the same ranges (33.3% positive correlations; 41.6% positive correlations): within soil pH values of 4.6–8.8, positive correlations can be found for different crop rotations (maize–wheat, asparagus–lettuce–tobacco, broad bean–tobacco, oilseed rape–tobacco, asparagus–lettuce–maize, broad bean–maize, and oilseed rape–rice) and field managements (also greenhouses) [77,83,85]. Negative correlations can also be found between soil pH 5 and 10 in other crop rotations (rice–wheat and celery–melon under a greenhouse) [21,25,86,89]. In one case, two different diversity indices were also found to be differently correlated with soil pH 8.5–8.8 in a watermelon cropping system with wheat and sunflower rotations, as the fungal Shannon–Wiener index displayed a positive correlation, while the fungal Simpson index displayed a negative correlation with soil pH [92].

The structure of the fungal community was the parameter most studied in correlation with soil pH, as explained by the composition, diversity, and interaction between individuals of the community. It was found to be mostly positively correlated with soil pH values between 5.3 and 6.5 (15.8% positive correlations) [36,37,69], but negative correlations (10.5% of found correlations) were also observed with more basic pH values (7.8–8.1) [35] or broader pH ranges (4.5–8.8) [39]. However, most studies (57.9% of found correlations) only stated the significant correlation between soil pH and the fungal community structure, within a pH range of 4.6–9, and for several crops, such as maize, wheat, and tobacco [28,38,53,70,71,73,74,75,76,87,88]. Even the fungi-to-bacteria ratio, calculated as the PFLA ratio in the soil of a strawberry farm [26], and the putative functional composition of fungal communities in wheat fields [93] were found to be positively correlated with soil pH ranges of 6.5–7.6 and 4.5–5, respectively.

Specific taxonomic categories can be correlated with soil pH too. *Ascomycota* phylum abundance has been mostly negatively correlated (60% of found correlations) with soil pH values between 5.6 and 8.1 in maize and intercropped pepper–maize fields [94,95,96], while a positive correlation occurred in watermelon–wheat and watermelon–sunflower fields within soil pH values of 8.5–8.9 [92]. Inside the *Ascomycota* phylum, the *Eurotiales*, *Helotiales*, and *Hypocreales* orders showed positive correlations with soil pH values between 5 and 7.5, although not always significantly [97,98]. Moreover, several genera, such as pathogenic *Alternaria*, *Chaetomium*, *Cladosporium*, *Dactylonectria*, *Macrophomina*, *Monographella*, *Mycosphaerella*, *Paecilomyces*, *Thielaviopsis*, and other non-pathogenic genera have been positively correlated with soil pH, ranging from pH 4.6 to 8.4 in one or more different studies [43,46,49,50,51,58,99,100,101,102,103,104,105]. However, genera such as *Alternaria*, *Chaetomium*, *Gibberella*, *Nigrospora*, *Stachybotris*, and *Tetracladium* were reported to have a negative correlation relationship with soil pH within the same soil pH range [49,50,51,58,99,100,102,106,107].

It must be noted that different communities or fungal genera were reacting differently to the same pH variations, as per abundance and/or diversity indices. When a specific fungal community is analyzed, the presence of different taxonomic groups may alter the response of the entire system. Moreover, external factors (e.g., crop rotation, field management, irrigation) can directly or indirectly influence the entire fungal community, as a whole and as single groups. This is why we also analyzed the responses of fungal genera and species, with particular regard for the pathogenic *Aspergillus* and *Fusarium* genera (Table 1).

The *Aspergillus* genus has been described to have a close relationship with soil pH [43], as it can affect the population (100% negative correlations) and its ability to produce toxigenic compounds (75% positive correlations). It must be reported that *Aspergillus* abundance in a wheat root rot-contaminated field in China was negatively correlated with soil pH values ranging from 6.5 to 7.2, though not significantly [100]. Samples from groundnut-cultivated fields in Ethiopia revealed that the highest population of *A. flavus* and *A. parasiticus* (≥500 cfu/g of soil) occurred in soil samples with a pH < 7, thus an acidic pH, while the lowest populations were highly correlated with soil pH ≥ 7 [2]. Regarding AF contamination, most studies reported that the correlation with soil pH was positive, in contrast with the previous statements for *Aspergillus* abundance and presence. In Kenya, it was reported that areas with slightly higher soil pH in the topsoil (6.03 vs. 5.86) can have a higher prevalence of AFs and contaminated maize samples [41]. Inside the gradient boosting machine (GBM) Iowa-centric model, soil pH was reported as one of the top two influences for AF contamination prediction with the 5 µg/kg AF threshold [108], and a positive correlation between AF levels and soil pH (between 0 and 50 cm in soil depth) in the hot–dry and hot–humid regions of Texas was found, according to the Ratkowsky–ARI nnet model [3]. Another gradient boosting decision tree (GBDT) model applied in Texas reported that soil pH, along with other soil physicochemical properties, is significantly correlated with aflatoxin contamination, although its correlation coefficient was relatively low [65]. This apparent contradiction between AF production and fungal growth suggests that soil pH differentially affects fungal growth and mycotoxin biosynthesis. However, in Pakistan, chili AF contamination in a recorded dry year (2019) was negatively correlated with soil pH, with soil pH values ranging from 7.3 to 7.9 [42]. This negative correlation was recorded for a more alkaline pH range than the range before (6.03–5.86 vs. 7.3–7.9), stating the possibility of a nonlinear relationship between soil pH and AF contamination.

The impact of soil pH on the *Fusarium* genus has been evaluated more than on the *Aspergillus* genus. This genus was observed as positively (16.7% of found correlations) and negatively (83.3% of found correlations) correlated with soil pH, although its abundance has been mostly negatively correlated with soil pH values between 5.5 and 8 [50,51,52,53,54,55,56,58,75,105,109]. The only study that reported a significant positive correlation between soil pH and *Fusarium* abundance reported a soil pH range of 4.5–5.5 [49], more acidic than the prior articles. Interestingly, the articles that reported a positive relationship between *Fusarium* and soil pH were based on a maize rotation system with different crops (wheat, bean, and ginseng) [49,103,110]. On the other hand, the articles reporting a negative relationship between *Fusarium* and soil pH implemented different treatments, such as mulching, and management techniques, such as continuous cropping, potting, and rotation cropping, involving maize, soybean, wheat, and other crops [52,53,54,55,56,57,109]. Thus, a negative relationship between soil pH and the *Fusarium* genus was found under several field management conditions and between sub-acidic to alkaline pH values. In particular, *F. graminearum* and *F. verticillioides* were reported as negatively correlated with soil pH [52,54,55]. Different studies have also negatively associated soil pH with *Fusarium*-related diseases, such as *Fusarium* head blight or root rot [1,111]. A few results also reported a positive correlation between soil pH and DON production [59]. In cups filled with soil samples at different soil moisture, soil texture, soil pH, and pathogen inoculum levels, soils at pH 6 showed more evidence of *F. graminearum* root rot in soybean plants among all treatment combinations compared with pH 8 [111]. In strawberry fields treated with either wheat straw mulching or plastic film, DON contamination was higher under the plastic film treatment, which was associated with a higher soil pH condition (pH 7.6–8.2) rather than the straw’s lower pH range (pH 6.3–7) [59], although DON production might have also been affected by the different treatment conditions. Soil pH also plays a role in the incidence of *Fusarium* head blight (FHB) in winter wheat, with slightly acidic soils (pH 5.5–6.5) being more conducive to the disease [1]. Thus, acidic pH values may appear more conducive to the disease, while slightly alkaline soils might be more correlated with mycotoxin production, possibly due to unfavorable conditions at higher pH levels. This is why soil pH was used and evaluated as one possible input (MI score > 65%) for the development of a robust machine learning (ML) model for predicting the within-field spatial distribution of FHB in winter wheat [1].

Still, not all studies agreed on considering soil pH as a key influencing factor for all fungal communities, since the pH range for fungal growth can be broad [112]. This could be the reason why different articles reported no statistically significant correlations between soil pH and the fungal community [27,98,104,113,114,115,116,117,118,119,120].

#### 3.1.2. Soil Water Content

Soil moisture or soil water content (SWC) is another important variable to take into consideration when studying the microbial community in the soil, as it describes the amount of water particles present and/or biologically available. SWC can influence the growth and composition of the microbial community directly [22], as well as through changes in the availability of soil nutrients [121].

SWC was the second most studied soil physicochemical property among the retained articles, with 46% of the research articles calculating SWC (Figure 2a). It has been mostly investigated through laboratory analyses, such as oven drying, weighing, use of a pressure plate apparatus, or desiccators (Figure 4b). SWC is mainly calculated as volumetric water content (VWC, %*v*/*v*), or the volume of water contained in a predetermined volume of soil (expressed as %), and as gravimetric water content (GWC, %*w*/*w*), or the mass of water per mass of dry soil (expressed in %). However, SWC can also be analyzed through other soil characteristics, such as soil water holding capacity (SWHC, %), depth of the first horizon (DRH, cm), or the water potential (MPa) in the soil. It can also be compared with rainfall and rain incidence in a given area (mm/year or m^3^/ha). To standardize the found results, VWC was considered the main unit of measure and converted from GWC, when possible, through soil bulk density. When the soil bulk density could not be defined, it was standardized to the average soil bulk density value of 1.33 g/cm^3^. If SWC was measured through other non-standard units, the initial units and values were maintained. For instance, DRH, showing redoximorphic features, was used to describe soil moisture conditions, with a value of 101 cm assigned to the profiles showing no redoximorphic features in the first 1 m of depth [11]. Some studies, however, relied on soil moisture meters, such as time-domain reflectometry (TDR) soil moisture meters (TDR-100) [20,122,123] and the TEROS 12 soil sensor (METER group GmbH, Pullman, Washington, DC, USA) [60], or weather stations with built-in soil humidity probes, such as the soil humidity sensor 10HS S-SMD-M005 (Onset Computer Corporation, Bourne, MA, USA) [123,124]. However, only three out of five articles specified the depth at which the soil sensors were placed to monitor SWC: 7, 10, and 22 cm [20,60,122].

Regarding the fungi, SWC was reported as an important factor influencing the fungal community and composition [21,27,28,62]. This influence was studied within different soil types (loamy sand or silt loam) [23], different added amendments (bentonite) [44] and different crop management conditions (mulching, fertilization, or irrigation regimes) [22,27,34,62,125]. Fungal abundance and richness mostly have a negative correlation (50% of found correlations) with SWC, as calculated between 12% and 24% (*v*/*v*) in maize fields and in a maize–wheat rotation [21,22,28,83]. Fungal diversity, on the other hand, mostly has positive correlations (83.3% of found correlations) with SWC in an SWC range between 9% and 27% (*v*/*v*), considering maize, wheat–maize–soybean, and wheat–oat as crop rotations [21,28,29,62,83]. Higher water availability might alter community composition positively, favoring the presence of multiple species and genera. It has also been hypothesized that drought conditions or water stress could drive fungal evolution [20]. In a tillage and crop rotation experimental maize field, the fungal communities present may have evolved to be drought-tolerant, suggesting a possible microbial biomass and composition adaptation [20].

As per soil pH, the effect of SWC on fungal genera might differ from one genus to another, also depending on the external factors involved. SWC was also found to be positively and negatively correlated with different fungal phyla, such as *Ascomycota*, *Basidiomycota*, and others [83,107,125]. The *Ascomycota* phylum abundance was found to be positively correlated with SWC in most studies (66.6% of found correlations), within 5–50% SWHC, but it was also found to be negatively correlated with SWC under higher SWHC (65–72%) [24,83,107,125]. The pathogenic genera *Sarocladium* and *Talaromyces*, along with other non-pathogenic ones, have been positively correlated with SWC in maize fields between 10% and 51% (*v*/*v*) [44,61,102]. However, the plant pathogenic genus *Alternaria* has also been negatively correlated with SWC between 10.6% and 10.5% (*v*/*v*) in maize fields [44]. The genera *Cladosporium*, *Gibberella*, *Macrophomina*, and *Penicillium* have also been negatively correlated with SWC between 10% and 51% (*v*/*v*) in maize and other crops or crop rotations [58,60,102].

Within fungal genera, *Aspergillus* and *Fusarium* have also been studied in relation to SWC. *Aspergillus* abundance has been positively correlated (75% of found correlations) with SWC between 6% and 31% SWHC in peanut fields and between 10.7% and 12.5% SWC (*v*/*v*) in maize fields [2,44]. Moreover, in pistachio experimental pots and fields contaminated by *A. flavus*, the percentage of atoxigenic *A. flavus* AF36 sporulated grains was higher at higher soil moisture levels (21.5–28.4% *v*/*v*) [45]. However, maize samples from semi-arid regions in Kenya (550–900 mm/year of rainfall) showed equal or higher *A. flavus* isolation rates compared with humid regions (800–1000 mm/year of rainfall), suggesting that low rainfall alone does not necessarily impact fungal occurrence [126]. On the other hand, disease incidence and AF production always showed a negative correlation with SWC (100% of found correlations) [3,9,10,126,127,128]. For instance, it was reported that prolonged soil moisture stress (85 to 100 days of drought) coupled with near-optimal soil temperatures (29 °C) can promote *A. flavus* and *A. parasiticus* infection and facilitate AF production in peanuts [10]. In maize fields in Kenya, contaminated grains and AF production were also mainly found in semi-arid regions [126]. Predictive models from Alabama, Texas, and Illinois indicate a negative correlation between AF production and SWC (0–31% *v*/*v*) in maize and peanut fields, confirming SWC as a key predictor of AF risk [3,9,65,127].

On the other hand, SWC showed opposite correlation dynamics with the *Fusarium* genus compared with *Aspergillus*. *Fusarium* abundance was found to be negatively correlated with SWC (83.3% of found correlations) between 10% and 22% (*v*/*v*) and between 17.8% and 27% (*v*/*v*) in maize fields and fields with maize-centered crop rotations [54,55,60,61,129]. However, disease incidence and mycotoxin production have been positively correlated with SWC between 16% and 27% (*v*/*v*) and between 18 and 33% (*v*/*v*) in maize, wheat, and strawberry fields [9,11,59,122,124,130,131]. A complex relationship with SWC and other environmental variables involved in *Fusarium* growth has been hypothesized to determine the severity of *Fusarium*-related diseases [122,123,130] and mycotoxin contamination in fields of wheat and triticale [11,124]. However, *Fusarium*-related diseases and mycotoxin contamination were found to be positively correlated with SWC (100% of found correlations). In particular, crown rot infection of wheat seedlings was found to be favored in moist soils, with a water potential between −0.3 and −0.7 MPa, while a low infection can occur when the water potential is less than −1.5 MPa [130]. Wheat seedling resistance was also reported to be more pronounced in limiting-water conditions [130]. In ginger fields, rhizome rot disease was accentuated by 29.9% (*v*/*v*) SWC during disease development [122]. The average DON contamination was also more pronounced in humid and less exposed sites in maize fields [11]. Regarding FB contamination, the gradient boosting machine (GBM) model analyzing FB contamination in maize fields in Illinois, USA, showed that FB contamination is positively correlated with SWC between 21% and 31% (*v*/*v*), representing the only predictive model found to investigate *Fusarium* mycotoxin contamination based on soil water content [9].

#### 3.1.3. Soil Electrical Conductivity

Electrical conductivity (EC) represents the capacity of a material to conduct electric current, or how well a material allows an electric charge to flow through it. In the soil, EC is influenced by and can influence the number of soluble salts in the soil water, and it is an indicator of soil salinity. That is why soil EC can affect the availability of soil nutrients [25] and can also reflect other soil properties, such as pH, moisture, or texture. EC is the third most measured physicochemical property among the retained articles (almost 23% of all measurements, Figure 2a). Similarly to soil pH and moisture, it is measured mainly through specific laboratory instruments, such as an EC meter (74% of EC measurements) (Figure 4c). In only 2 out of 22 articles that measured soil EC was in-field soil sensing technology, specifically TDR, used to monitor crop rotation systems with wheat, maize, and soybean [60,62]. Eleven percent of EC recordings were also taken from online databases, such as the USDA NRCS-WSS database and the USDA NASS-CDL (Appendix B) [3,9,108].

Following soil pH and moisture, soil EC was reported as an important environmental factor that is significantly correlated with the fungal community structure [30,67], abundance [68,86], and diversity [30,86], even though it may be an indirect influence [25]. In particular, fungal community abundance was positively correlated (100% of found correlations) with soil EC between 100 and 1200 µS/cm in different crop management (open field and greenhouse) and crop rotation (rice–wheat and wheat–maize) systems [25,67,91]. On the other hand, fungal richness and diversity were negatively correlated (100% of found correlations) with soil EC, as studied between 160 and 6700 µS/cm in maize fields and salt-contaminated fields [30,86].

Specific fungal phyla have also been correlated with soil EC [86,107]. Positive correlations were found between soil EC and the pathogenic and non-pathogenic genera *Chaetomium*, *Chrysosporium*, *Cladosporium*, *Penicillium*, *Trichoderma*, and *Verticillium,* mostly within the ranges of 180 to 645 µS/cm and 3900 to 5500 µS/cm, while negative correlations were also found with other genera, such as *Ilyonectria*, *Neurospora*, *Stachybotris*, *Talaromyces*, and *Tetracladium* in the same soil EC ranges [46,102,132].

Not many articles investigated the correlation between soil EC and fungal communities or genera, including the *Aspergillus* and *Fusarium* genera. Only one article pointed out a negative correlation between *Aspergillus* abundance in soil and soil EC between 3900 and 5500 µS/cm [46]. The soil EC influence on *Fusarium* abundance and disease incidence was more discussed in the retrieved articles. Along with other parameters, the disease incidence of *Fusarium* maize stalk rot and symptomatic ears was positively correlated with soil EC (20–830 µS/cm) in maize fields treated with sewage sludge over a 5-year experiment [133]. A positive cause-and-effect correlation between soil salinity, expressed as soil EC, was also hypothesized for wheat crown rot in saline soils [134]. However, in a long-term crop rotation field experiment involving several crops (maize, soybean, wheat, pea, sunflower, and oat), the population density of *F. oxysporum* did not show any significant correlation with soil EC [60]. The soil EC range, on the other hand, was not reported.

Even though soil EC was present in the few attempts made to model mycotoxin contamination using soil properties, its influence was not emphasized or pointed out in the articles’ discussions [1,3,9,108].

#### 3.1.4. Soil Temperature

Soil temperature, measured at a specific depth, is a crucial factor influencing various biological, chemical, and physical processes within the soil. It is connected to soil moisture and can affect the growth and development of microorganisms and plants. Soil temperature is the least measured soil physical property among the retained articles (almost 12%, Figure 2a). However, in contrast with the already-described soil properties, the main measurement tools for soil temperature are real-time solutions, such as data loggers or soil thermometers (Figure 4d). Data measurements were mostly taken every 10 min to every hour through soil temperature sensors (e.g., TEROS 12 soil sensor) and then stored inside a data logger and visualized according to the study [20,26,60,62,63,74,91].

Soil temperature was reported as a substantial predictor of fungal abundance and diversity [28,62,135]. Fungal diversity and abundance were mostly negatively correlated with soil temperature between 15 and 27 °C and between 30 and 43 °C [28,62,91,136], although not many studies were found to clearly analyze or state the correlation direction between soil temperature and fungal communities.

Not many phyla, orders, or genera were separately investigated for correlations with soil temperature, except for *Aspergillus* and *Fusarium*. In peanuts, aflatoxigenic fungal invasions and AF production are positively correlated (100% of found correlations) with soil temperature and can occur within an optimal soil temperature range between 25 and 30 °C [10,126,137]. Furthermore, the percentage of *A. flavus* S-type strains that are known to be high AF producers [126] was found to increase linearly with soil temperature. Ranging from 16 to 32 °C, the percentage of *A. flavus* S-type strains was recorded at 2.54 cm of soil depth within four different crop rotations (maize–cotton, maize–sorghum, cotton–sorghum, and sorghum–soybean) [47]. According to a gradient boosting model for AF contamination risk in maize, soil temperature was calculated as one of the five most relevant predictors (out of the initial 102): in particular, soil temperatures between 18 °C and 27 °C in May and June are strongly associated with increased AF risk. Soil temperatures between 24 and 27 °C were positively correlated with higher risk classes, while temperatures between 18 and 24 °C were more associated with low and moderate risks [48].

Regarding *Fusarium*, high fungal abundance and disease incidence can also be linked to high soil temperatures, particularly between 16 and 36 °C [60,62,122]. However, the data were insufficient to hypothesize a clear correlation. In soils treated with anaerobic soil disinfestation (ASD) treatments, the *F. oxysporum* f. sp. *lycopersici* population was significantly lower under higher temperatures (25–35 °C) than under lower temperature ranges (15–25 °C) [63]. In ginger fields, *Fusarium* rhizome rot reached up to 92.1% of disease incidence, possibly due to a congenial microenvironment during the development period regarding soil temperature (28.9 °C) and soil moisture (29.9% SWC *v*/*v*) [122]. In a long-term crop rotation field experiment involving several crops (maize, soybean, wheat, pea, sunflower, and oat), the population density of *F. oxysporum* was positively correlated with soil temperature, although the soil temperature data were not reported [60].

### 3.2. Soil Sensing and the Use of Continuous Sensors

Soil physicochemical properties, such as pH, electrical conductivity (EC), soil water content (SWC), and temperature, have been qualitatively analyzed to explore their correlation with the fungal community in the soil. Different approaches have been proposed to obtain more precise field data quickly and easily, including soil sensing. A soil sensor can be described as a tool or device used to measure various physical and chemical properties of the soil [138]. Proximal, remote, and in-field continuous soil sensing are new methods for directly or indirectly monitoring important soil properties, with little or no field disturbance. Proximal soil sensing uses hand-held or moving sensors close to or in direct contact with the soil, relying on optical, geophysical, electrochemical, and mechanical techniques to measure relevant properties [139]. Remote soil sensing uses space-borne microwaves and optical sensors to map soil characteristics with high resolution [140]. On the other hand, in-field continuous soil sensing (or soil monitoring) relies on ongoing “smart” sensors that are directly placed in the ground and can keep track of soil parameter fluctuations [141]. Each soil sensor follows a specific technique or theoretical method to measure each soil property, giving the sensor specific characteristics or specifications. In Figure 5, the percentages of measurement techniques used by the retrieved soil sensors are summarized. Each quoted technique is further explained in Appendix A.

Research articles were also qualitatively examined for their methodology used to gather in-field soil data (Figure 4) and their application of in-field sensors to measure those soil physicochemical properties. Any technique that was not specified was considered not defined, and the calculated percentage value of each used technique was evaluated as a qualitative trend. Mycotoxin predictive models found within the research were also analyzed for their implementation of soil properties. How soil data were acquired for model training was investigated. All retrieved predictive models gathered soil data through online databases and surveys, particularly using the Food and Agriculture Organization of the United Nations (FAO) Harmonized World Soil Database (HWSD), the United States Department of Agriculture (USDA) Natural Resources Conservation Service (NRCS) Web Soil Survey (WSS), the USDA National Agricultural Statistics Service Cropland Data Layer (NASS-CDL), and the Google Earth Engine (GEE) [3,9,41,65,108]. The description of each database can be found in Appendix B.

#### 3.2.1. Soil Water Content (SWC) Sensing

SWC is particularly investigated when monitoring soil health, as 95% of the retrieved soil sensors are fabricated to measure this soil characteristic (Figure 2b). Soil moisture levels can help maintain suitable conditions for field ecology [142,143] and final yields [144], as they can be directly regulated through controlled irrigation regimes. To monitor soil moisture in the field, SWC sensors can be grouped according to their working mechanisms (Appendix A.1) [138,144,145,146,147].

Optical SWC sensors are mainly utilized as remote or proximal sensing tools. None of the retained in situ soil sensors employed this technology in the articles on fungal presence, since the search mainly focused on in-field solutions.

On the other hand, neutron-based sensors were deployed in the field to monitor soil moisture [148]. This fairly new technology was chosen for two joint field campaigns from May to July 2019 in the 1 km^2^ pre-alpine area of the Rott headwater catchment (Landsberg, Germany), and then it was implemented from September to November 2020 in the 0.4 km^2^ Wüstebach catchment (Eifel mountains, Germany) by German research institutions. By deploying 24 and 15 Cosmic Ray Neutron (CRN) sensors, the scope of that study was to explore the use of dense stationary CRN networks for monitoring spatiotemporal soil moisture dynamics in catchments [146]. Another CRN system was successfully deployed in two experimental fields in Potsdam, Germany, and in Lagosanto, Italy, to test the capabilities of the newly developed Finapp probe (Finapp s.p.a., Padova, Italy) compared with two commercial alternatives (CRS-1000, Hydroinnova. Albuquerque, New Mexico; CANBERRA probe, Canberra Industries, Meriden, CT, USA) and another point-based soil moisture sensor (5TE, METER Group GmbH, Pullman, WA, USA) [149].

Apart from these solutions, electromagnetic sensors are currently the most widely used soil moisture sensing technology. A proximal example of an SWC sensing technology based on electromagnetic fields is electromagnetic induction (EMI) [144,145]. However, focusing on in situ continuous sensors, other types of electromagnetic sensors are the so-called resistance-based sensors or the permittivity-based sensors. Some commercial resistance-based SWC sensors are the YL100 and YL69 soil moisture probes [138,150,151,152,153,154]. Pure capacitive sensors are also implemented in the fields through an Internet-of-Things (IoT) approach. The HD3910.1/HD3910.12 probes (Senseca Germany Gmbh, Regenstauf, Germany) are an example of pure capacitive sensors, which can measure SWC with minimum soil disturbance and different communication protocols (RS485, SDI-12, Analog), although they are best suited for measurements in small volumes. Capacitive technology has been implemented and explored in different ways between commercial sensors, with several adjustments and improvements, leading to the development of time-domain reflectometry (TDR), time-domain transmittometry (TDT), frequency-domain reflectometry (FDR), and many other sensing technologies (Appendix A.1) [138,141,144,145,155,156]. Several sensors have been developed adopting TDR technology, and they are now commercially available for measuring SWC along with other physicochemical properties, such as soil EC, soil pH, and soil temperature [122,150,155,156,157]. For example, the JXCT sensor (Weihai Jingxun Changtong Electronic Technology Co., Weihai, Shandong, China) can measure soil pH, temperature, moisture, EC, and NPK, with an RS485 interface and Modbus RTU protocol [150,158]. The TDR 315/310 H sensors (Acclima Inc., Meridian, ID, USA) can be used to measure soil temperature, moisture, and EC, with an SDI-12 communication protocol [155]. The TDT Aquaflex SI.99 soil sensor (Aquaflex soil moisture sensors, Onfarm Data, Middleton, Christchurch, New Zealand) uses TDT technology for measuring soil temperature and moisture [159]. Moreover, FDR sensors are well implemented in the literature and commercialized [142,155,160,161,162,163,164,165]. For instance, the MEC10 sensor (Dalian Endeavor Technology Co., Dalian, China) can measure soil temperature, moisture, and EC, with an RS485 interface and Modbus communication protocol [165]. The Drill and Drop sensor probe by Sentek (Sentek Sensor Technologies, Stepney, South Australia, Australia) uses capacitance/FDR technology to measure soil temperature, moisture, and EC, and it can be connected via RS232, RS485, SDI-12, and Modbus communication protocols [155]. The Drill and Drop probe (Sentek Sensor Technologies, Stepney, South Australia, Australia) is available from 30 to 120 cm in length, with sensors placed every 10 cm to measure soil temperature, moisture, and EC at different depths to obtain a complete view of the soil profile and state in the short or long term.

Several newly developed solutions are surfacing from the literature. For instance, inductively coupled capacitive soil moisture sensors were developed on a biodegradable paper-based substrate, and they are able to respond to soil moisture changes over longer periods of time compared with other commercial solutions [166].

#### 3.2.2. Soil Electrical Conductivity (EC) Sensing

Soil EC, as a direct consequence of soil salinity, is frequently measured along with soil water content (SWC), expressed as the dielectric constant or permittivity of the soil εa. Bulk EC, defined as in the EC of the bulk soil (a mixture of soil, water, and air), is the value measured by all conductivity sensors. Empirical or theoretical equations can be implemented in EC sensor algorithms or programming to determine pore water EC and saturation extract EC from bulk EC values.

Forty-one percent of the retrieved soil sensors measured bulk EC, and all of them also measured SWC. An explanation can be found regarding the way soil sensors technically measure bulk EC: a pair of electrodes introduces an electric current into the soil, while other receiving pairs of electrodes measure the voltage attenuation. This method has many similarities with resistance-based soil sensors designed to measure SWC. This is also why other electromagnetic technologies (TDR, FDR, and impedance; Appendix A.1) have been commercialized and deployed to measure bulk EC and SWC in the field [122,150,155,157,161,165,167,168,169]. The 5TE soil sensor (METER group GmbH, Pullman, WA, USA) is an example of an FDR/capacitance-based soil sensor that measures soil bulk EC, along with soil moisture and temperature, with the possibility of integrating an SDI-12 communication protocol [155,161,167]. The CS655 and CS650 soil sensors (Campbell Scientific Inc, Logan, UT, USA) are based on TDR technology for measuring SWC and bulk EC, while they also measure soil temperature [155,157,168]. The WaterScout SMEC 300 soil sensor (Spectrum Technologies Inc, Aurora, IL, USA) is a resistance-based bulk EC sensor that is also able to measure SWC and soil temperature [151,170].

#### 3.2.3. Soil Temperature Sensing

Several sensors have been developed to monitor soil temperature (57% of all retrieved sensors), such as thermocouples, resistance temperature detectors (RTD), thermistors, diodes, and band-gap temperature (SBT) sensors (Appendix A.2) [138,144,145].

One of the most common sensing technologies for temperature found in soil sensors is the thermistor. The ThetaProbe ML3 soil sensor (Delta-T Devices Ltd., Cambridge, UK) has a built-in thermistor to measure soil temperature, while also monitoring soil moisture and soil electrical conductivity (EC) [151,155]. The TDR 315 H and TDR 310 H soil sensors (Acclima Inc, Meridan, ID, USA) use thermistors for measuring soil temperature, along with TDR for soil moisture and soil EC [155]. One example of an RTD sensor is the Pt100 probe implemented by the TP32MTT.03.A/B sensors (Senseca Germany Gmbh, Germany), which can measure the air and soil temperatures through a double-sensing probe. Diodes can also be implemented in soil temperature monitoring. An example of K-factor diodes is the SLT5006 soil sensor (Murata Manufacturing Co., Kyoto, Japan), which can also measure soil moisture and soil EC [171]. The main advantage of an SBT sensor is that it can be integrated into a silicon Integrated Circuit (IC or “chip”) at a very low cost, with better monitoring performance [144]. The Belgian experimental Niphargus sensor and data logger [172] implements SBT ADT7410/ADT7420 probes (Analog Devices Inc., Wilmington, MA, USA) to monitor the temperature of local natural processes in the soil using an I2C communication protocol.

#### 3.2.4. Soil pH Sensing

Soil pH can be monitored through in situ soil sensing, although fewer available solutions have been found, since only 3% of the retrieved sensors measured soil pH. Methods for measuring soil pH include colorimetric or photometric approaches, conductometric or potentiometric approaches, and acoustic approaches (Appendix A) [144,145].

Although traditional conductimetric sensors are not suitable for in situ measurements, new technological advancements have been proposed to enable in situ soil pH conductometric monitoring, such as potentiometric pH sensors or acoustic sensors (Appendix A) [144,145]. Some known commercial examples of potentiometric sensors are the solid dielectric pH probes with a polytetrafluoroethylene (PTFE) liquid junction, made to last longer and be almost maintenance-free. The S-pH-01-A sensor (Seeed, Shenzhen, China) and the ZSPH soil pH sensor (ZATA IOT, Hong Kong, China) are solid dielectric pH sensors that can connect through a MODBUS RS485 communication protocol to data loggers or other output devices. In particular, the S-pH-01A sensor also has built-in automatic temperature compensation (ATC) to automatically compensate for possible temperature errors. Acoustic-based pH sensors are highly sensitive to pH but more complex and more sensitive to noise [144,145].

Some alternatives for soil pH sensing include ion-selective field effect transistors (ISFETs) and magnetoelastic pH sensors [138,144,145,173]. Unfortunately, the only commercially available soil pH sensors described within the literature search were the JXCT [150] and Atech 8 in 1 [174] models, the measurement methodology of which is not clearly described by the manufacturers. Additionally, to save money on more expensive probes, it is also possible to fabricate a custom printed soil pH sensor, which can include the following: an Alizarin (pH-sensitive redox compound 1,2-dihydroxyanthraquinone) composite working electrode, coated with a Nafion membrane; a salt-membrane-coated reference electrode; and a simplified two-electrode architecture, allowing for more devices to capture in-field continuous information [175].

## 4. Discussion

This systematic literature review examined how key soil properties (pH, EC, SWC, and temperature) are measured and their influence on fungal communities—particularly *Aspergillus* and *Fusarium* spp.—and how these variables are incorporated into mycotoxin predictive modeling. Despite the influence of other emerging variables, such as field management and block trials, and despite the limitations of the latest time range considered (2020–2025), the evidence highlights soil properties not merely as background variables but as active modulators of host–pathogen–environment interactions, even if they are rarely included in modeling.

Soil physicochemical properties can modulate the occurrence and pathogenic behavior of fungi directly or indirectly. Soil pH affects nutrient availability, microbial activity, physiochemical soil functions (e.g., cation exchange capacity, redox potential), and broader multi-trophic interactions relevant for plant growth and yield [1,3,108]. It can also influence the equilibrium between pathogens and their microbial antagonists in the soil [1,111]. While soil pH is usually studied and discussed alone, SWC and soil temperature are often correlated and studied together. High soil temperature and drought stress alter plant physiology, reducing growth and increasing susceptibility to fungal attacks. These conditions also favor the growth of *A. flavus* and *A. parasiticus* and stimulate AF production [3,10]. A significant increase in proline, an AF production enhancer, and the inhibition of phytoalexins, antimicrobial compounds produced by some plant species, were observed due to drought stress [10]. Under field conditions, peanut seeds were most susceptible to *A. flavus* invasion and AF contamination at water activity levels of 0.90–0.95, since higher water activity enhances phytoalexin activity, inhibiting fungal colonization [137]. Excessive drought can also crack peanut pods and kernel seed coatings, establishing new entry points for aflatoxigenic fungi [10]. High soil temperatures can enhance moisture loss from peanut kernels and reduce their water activity level, favoring fungal infection [2].

In maize, a positive relationship between high early-in-the-year soil moisture and following AF outbreaks was observed [3]. Excessive early-season soil moisture can lead to shallow root development, reducing the plant’s ability to access deeper water later in the season and increasing susceptibility to drought stress [3]. The crops will then experience increased drought stress and become more susceptible to AF contamination [3]. Conversely, higher soil moisture often favors the growth of mycotoxigenic *Fusarium* spp., such as *F. graminearum*, on crop residues, promoting inoculum production and spore maturation [11,54]. Furthermore, increased water availability can influence not only the speed of spore colonization and the survival of the fungus in the soil or on plant debris but also the development of antagonistic or competing microorganisms and the interactions among them [11]. Water availability, including SWC, is critically important for these biological processes [11].

Even soil salinity, described and calculated as soil EC for *F. pseudograminearum* in wheat, may reduce the plant’s ability to resist or delay fungal infection [134]. The propagule density of *A. flavus* is negatively affected when the daily average soil temperature is below 18 °C and above 30 °C [2,47], and the highest population density was recorded at a 25–30 °C daily soil temperature [2]. However, the population of *A. flavus* and the relative density of the S-strain were reported to increase linearly with soil temperature [2,137], thus indicating that the different strains of *A. flavus* may have different ecological requirements.

However, a distinction between the soil ecological requirements for mycotoxigenic fungal growth and the conditions needed for mycotoxin production was pointed out, as efficient mycotoxin production is often expected under stressful conditions for fungi [3,42,59]. *Aspergillus flavus* thrives at around 7.5 pH, within a range between 3.5 and 8 [108,176,177], while AF production increases at more acidic pH levels [3,41]. For example, *F. graminearum* root rot in soybean was more severe at pH 6 [111], whereas DON contamination in strawberries under wheat straw mulching with conventional planting rows or under black polyethylene film was higher at pH 7.8 than 6.7, indicating that the pH effect of *Fusarium*-related diseases can vary by crop and species [59]. Regarding SWC, drought or low soil moisture conditions are mainly reported as positively correlated with AF contamination [3,10,42,126,127]. Instead, different studies disagree on whether *Fusarium*-related diseases and mycotoxin production are positively or negatively influenced by SWC. *Fusarium* infection can be favored by high soil moisture, which is reported as one of the ideal conditions for fungal growth and pathogenicity [11,59,122,123,124,130]. However, several studies reported low SWC as more correlated with *Fusarium* presence [54,55] and even suggested sowing the crops in early winter to increase the SWC levels and affect the development of *Fusarium* wilt [129]. Taken together, these findings highlight that environmental conditions favoring fungal establishment and proliferation may differ substantially from those triggering mycotoxin biosynthesis. Nevertheless, soil properties can be useful to evaluate the incidence of fungal diseases and/or mycotoxin contamination in the field. Soil properties have been added to regression or predictive models in recent years [1,2,3,9,48,108], and all those models highlighted the significant role of soil characteristics in relation/prediction of mycotoxin outbreaks. In Iowa, integrating soil properties into AF models markedly improved predictive performance, with accuracies of 96.8% (20 µg/kg threshold) and 90.3% (5 µg/kg threshold) [108].

Machine learning (ML) models trained with field-specific soil characteristics and satellite data demonstrated that high-resolution soil information substantially improves the prediction of *Fusarium* head blight in wheat [1]. These few examples are only the starting point to improve predictive modeling, considering new and important variables that are already detected in the field. However, more reliable and precise data are needed directly from the area considered. Most retrieved predictive models have taken data from online surveys, databases, or satellites [3,9,48,108]. Only for the prediction of winter wheat *Fusarium* head blight in Lithuania was an in-field solution for measuring EC used, although it consisted of a mountable apparatus (EM38-MK2 apparent electrical conductivity scanner, Geonics Ltd., Mississauga, ON, Canada), which can measure apparent EC through electromagnetic conduction only when mounted on a moving vehicle and carried across the field [1]. Satellites and online databases can provide large datasets, but their outputs benefit from verification and standardization using locally collected soil data to capture fine-scale variability and improve model accuracy [48]. Thus, to have more reliable and precise soil information, in-field continuous solutions must be considered.

Soil sensors can be an incredible tool for continuously monitoring different soil physicochemical properties. However, to decide which sensor model to install in the field, several variables must be taken into consideration: the theoretical measurement method, the capability to measure different properties, cost-effectiveness ratio, field positioning, the presence of manufacturer calibration, communication protocols, and external digital connections. Moreover, those devices can be continuously exposed to various external interferences, making them susceptible to reading alterations, with a small error percentage always present in working electronic devices. Too high or too cold temperatures, heavy rains, strong winds, high soil salinity, and other environmental stresses can produce higher errors in the final readings, leaving behind uncertainty in the data. To minimize such errors, it is essential to analyze the soil context and calibrate sensors accordingly. These sources of uncertainty should not be regarded as limitations per se but rather as design constraints that must be explicitly addressed when deploying soil sensors under field conditions. Manufacturer calibration can be sufficient initially but may not be enough in the long run.

Soil temperature, salinity, texture, moisture, and organic matter can influence the final recordings of the sensor and the whole study [163,170,178,179,180,181,182,183,184]. For instance, temperatures within the low-to-medium room temperature range (<25 °C) corresponded to increased soil moisture sensor outputs for the SMEC 300 sensor models, with heightened fluctuations and outliers for temperatures exceeding those limits [170]. For the same sensor, a linear increase in raw output was also observed with varying salinity in silty clay and silty soils, while sandy soils exhibited a higher deviation from the initial calibration equation [170]. As for Sensoterra B.V. sensors, the effect of salinity on the final performance in four soil types (glass beads, sand, loam, and clay loam) was also greater for sandy soils compared with sandy and clay loams, while the effect of soil EC and temperature can affect sensor readings [178]. EC-5 sensors were also tested to measure the SWC of specific soil types, such as sandy soils, and temperature effects should be considered, particularly when the water content is measured at near-surface depths with great diurnal temperature fluctuations [163,185]. Even soil organic matter had a significant effect (*p* < 0.05) on the precision and accuracy of the commercially available 10HS soil water content monitoring sensor, resulting in an underestimation of the SWC at the dry water content range (<5% *v*/*v*) while overestimating the SWC at the relatively higher water content range (>5% *v*/*v*) [179]. This is why many studies have critically analyzed the initial calibration and characteristics of the available sensors [151,164,169] and improved them within their specific field or soil types, considering different external variables [163,178,179,180,186,187]. Several studies have also documented sensor-to-sensor variability, an inherent output difference between identical devices [163,170,178,188] and an innate output variability between the same type of tool or device.

Another variable that must be taken into consideration when deploying an in-field sensor is the external surroundings. Obstacles (e.g., trees, buildings) might interfere with the connection between the gateway and the sensor station or device if certain communication methods are involved. The location of the gateway, the sensor station, and the distance between them are important to take into consideration when placing the device(s). Even the charging method of the device and/or station must be chosen. Solar- or battery-powered stations are two possible methods of coping with charging limitations: a solar-powered station does not require much assistance; however, it can be fragile, and it cannot charge on most days. In contrast, a battery-powered station can be more stable over time, while batteries must be changed when power is low. Nonetheless, the economic side must also be considered. In-field soil sensors can cost $10 to $2000 and more depending on the reliability, sensitivity, durability, and overall production quality of the components.

Therefore, the selection of different soil sensing devices must be made with caution, particularly when a sensing station has multiple sensing devices. Applying a sensor combination system can minimize errors between different sensors and within a specific location [170,189] while recording different data. These sensor systems are also useful from an Internet-of-Things (IoT) perspective, when several multi-purpose sensors are connected together and to the final user through a digital platform to facilitate the storage and visualization of soil time series [190,191,192]. An IoT approach [145,150,191,193,194] or a wireless sensor network (WSN) [152,195,196,197] can help calibrate management practices, such as irrigation regimes, or predict crop productivity and health according to real-time needs and soil fluctuations [143,150,153,154,158,192,193,198,199,200,201,202,203,204,205,206,207,208]. An IoT approach can also be applied to monitor vast areas, such as regions or entire nations, with the use of carefully deployed weather and soil sensing stations, similar to the FESSTVaL experiment [209]. These devices can be connected to data loggers [210] and transmit the final data to the user, who can then consider the obtained datasets for further management decision-making or research [150,193,204,211,212,213]. Moreover, “smart” sensors and data loggers can be coupled with AI to improve decision-making and the final quality of the data [145,190,192,199,203,204,214,215,216]. Within this framework, eXplainable AI (XAI) techniques can be useful and important tools for making more transparent decisions and better understanding which factors, and why, have a specific influence on the main variable [217]. The integration of IoT with AI (namely AIoT) could represent the next step in supporting effective management strategies to reduce water losses, increase yields, and protect crops against early signs of disease, alongside end users [218].

Even though emerging technologies, including MEMS sensors, nanosensors, and biodegradable platforms, are rapidly advancing and enabling the in-field assessment of key physicochemical properties [154,158,219,220], substantial research is needed to expand and spread such in-field solutions. Micromechanical system (MEMS)-based sensors or nanosensors may provide real-time monitoring at a microscopic scale, enhancing hardware durability and accuracy [145]. Biodegradable sensors may overcome sustainability issues within the industry, using different encapsulating materials such as wood or beeswax [145,151].

## 5. Conclusions

Key soil properties, such as pH, EC, SWC, and temperature, can shape fungal communities. Pathogenic populations, including Aspergillus and Fusarium genera, are also affected by these soil characteristics, which can alter their pathogenic ability and mycotoxin production. Thus, soil properties are starting to be included in mycotoxin predictive modeling, and the first results are promising from the model robustness and prediction reliability perspective. However, soil data collection in the retrieved articles was mainly based on laboratory techniques or, in the case of predictive models, satellites and online databases. The use of these techniques and tools is efficient, considering the large amount of data acquired in a short period of time, but it is not sufficient for depicting the complete and current state of the field. The use of in-field continuous soil sensors can help acquire local reliable data to (i) train newly developed and/or existing predictive models to boost their sensitivity, robustness, and predictive power; (ii) standardize satellite data, which lack fine-scale resolution and can miss local variabilities; and (iii) use the obtained information to program field management options within an IoT perspective, enabling prevention rather than protection.

The shift towards IoT-based mycotoxin prediction enables a transition from reactive responses to anticipatory, risk-based decision-making at the field scale. Since farmers can check the state of the field in real time using multi-purpose weather stations and change irrigation or fertilization treatments, the digital platform (e.g., a mobile application, a website) could also be integrated with prediction risk alerts for potential mycotoxin contamination. When the risk threshold is exceeded, farmers can immediately take all possible actions to mitigate the predicted risk. The IoT scenario cannot be carried forward without the implementation of suitable soil sensors and advanced solutions. The advancement possibilities in field sensors are considerable, but issues connected to external or environmental factors and cost-effectiveness should be seriously considered. The right sensing station should be chosen carefully and placed in the correct location while being constantly monitored so that any issues can be resolved. Moreover, data management could be problematic, as there is a substantial amount of data that needs to be monitored and analyzed afterward. Nevertheless, the state of the art regarding commercialized and self-made soil sensors shows a thriving field, with new technological and technical possibilities explored daily to satisfy natural variability and farmers’ needs. Their implementation in the field should be further explored to find the missing link between field management, pathogen prevention, and a data-driven, IoT-enabled future for fungi and mycotoxin risk management.

## Figures and Tables

**Table 1:** Summary table reporting the positive or negative correlations between soil physicochemical properties (pH, soil water content, electrical conductivity, and temperature) and the abundance and/or mycotoxin production of *Aspergillus* and *Fusarium* genera. Symbol + indicates a positive correlation, while the symbol – indicates a negative correlation within a given range. Undetermined correlations or ranges are reported as “nd”, while not correlated results are reported as “nc”. Statistically insignificant but reported positive or negative correlations are not considered in the table. Each soil property is reported with a standardized unit of measure when possible.

Soil Physicochemical Property	Range	*Aspergillus* Genus Correlation (%)			
		Abundance		AF ^a^ Production	
pH	5.8–6	+	[41]		
	7–7.9	–	[2,42]	nd	
	8–8.4	+	[43]		
Soil Water Content	0–31%VWC ^b^	+	[44,45]	–	[3,9]
	6–31%WHC ^c^	+	[2]		
Electrical Conductivity (µS cm^−1^)	3940–5520	–	[46]	nd	
Temperature (°C)	16–32	+	[47]	+	[10,48]
		***Fusarium* genus**			
		**Abundance**		**DON ^d^** **Production**	
pH	4.6–5.3	+	[49]		
	5.5–8.1	–	[50,51,52,53,54,55,56,57,58]	+	[59]
Soil Water Content (%VWC)	10–13	+	[44]		
	10–22	–	[54,55,60,61]	+	[59]
Electrical Conductivity (µS cm^−1^)	nd	nc	[60]	nd	
Temperature (°C)	15–35	+/–	[62,63]	nd	

^a^ Aflatoxin; ^b^ volumetric water content (%); ^c^ water-holding capacity (%); ^d^ deoxynivalenol.

## Data Availability

The original contributions presented in this study are included in the article/Supplementary Materials. Further inquiries can be directed to the corresponding author.

## Supplementary Materials

The following supporting information can be downloaded from https://www.mdpi.com/article/10.3390/s26134044/s1, Figure S1. Circular tree map of retrieved article journals for (a) soil properties and (b) in-field soil sensors’ state of the art.; Figure S2. Network of retrieved author keywords for (a) soil properties and (b) in-field soil sensors’ state of the art.; Figure S3. Word cloud of retrieved author keywords for (a) soil properties and (b) in-field soil sensors’ state of the art.; Table S1. Measuring methodologies of in-field soil properties by retrieved articles; Table S2. Measuring methodologies of soil sensors retrieved by literature research. Table S3: PRISMA 2020 Checklist used in the current systematic review study. This systematic review protocol was registered in the Open Science Framework with the following Registration DOI: https://doi.org/10.17605/OSF.IO/EF6DB.

## Abbreviations

The following abbreviations are used in this manuscript:

FAO-HWSD	Food and Agriculture Organization—Harmonized World Soil Database
NASS-CDL	National Agricultural Statistics Service—Cropland Data Layer
NRCS-WSS	Natural Resources Conservation Service—Web Soil Survey
FESSTVal	Field Experiment on Submesoscale Spatio-Temporal Variability in Lindenberg
ISFET	Ion-Selective Field Effect Transistor
AFB1	Aflatoxin B1
AFB2	Aflatoxin B2
AHFO	Electrically Generated Heat Pulse
AIOT	Artificial Intelligence Internet of Things
GBDT	Gradient Boosting Decision Tree
IARC	International Agency for Research on Cancer
MEMS	Micromechanical Systems
PCoA	Principal Component Analysis
PLFA	Phospholipid Fatty Acid
SWHC	Soil Water Holding Capacity
USDA	United States Department of Agriculture
ATC	Automatic Temperature Compensation
CRN	Cosmic Ray Neutron
DON	Deoxynivalenol
DRH	Depth of the First Horizon
DTS	Distributed Temperature Sensor
EMI	ElectroMagnetic Induction
FDR	Frequency-Domain Reflectometry
FHB	*Fusarium* Head Blight
GBM	Gradient Boosting Machine
GEE	Google Earth Engine
IoT	Internet of Things
NIR	Near-InfraRed
SBT	Band-Gap Temperature
SWC	Soil Water Content
TDR	Time-Domain Reflectometry
TDT	Time-Domain Transmittometry
RTD	Resistance Temperature Detector
ZEN	Zearalenone
xAI	EXplorative Artificial Intelligence
WSN	Wireless Sensor Network
AF	Aflatoxin
AI	Artificial Intelligence
EC	Electrical Conductivity
FB	Fumonisin
ML	Machine Learning
OT(A)	Ochratoxin (A)

## Appendix A

### Appendix A.1

To monitor soil moisture in the field, SWC sensors can be grouped according to their working mechanisms into optical sensors, thermal sensors, neutron-based sensors, and electromagnetic sensors [138,144,145,146,147].

Optical sensors are usually based on visible and near-infrared (NIR) spectrophotometry, making them tools for remote or proximal sensing. As the moisture absorption bands exist in the NIR wavelength range (1450, 1940, and 2950 nm), when the SWC changes, the molecular absorption of light by SWC changes accordingly, resulting in a reflection spectrum variation that can be detected and converted to the corresponding moisture value [138,144].

Thermal sensors work on the heat-storing capacity of the soil, which is highly dependent on SWC. In a dual-needle heat-pulse probe, one needle is used as the heater, and the other is the temperature measurement device. The heater needle sends a heat pulse into the soil over time, which is recorded by the receiver needle. The maximum temperature difference recorded, which is dependent on the surrounding SWC due to a more efficient heat dissipation in wet conditions [219], is used to calculate the soil heat capacity and SWC. The same concept has been applied to fiber optics for temperature and SWC measurements. Fiber optic distributed temperature sensors (DTSs), namely, actively heated fiber optics (AHFO) or passive DTS, measure the SWC through the temperature change after the application of an electrically generated heat pulse (AHFO) or in response to net solar radiation (passive DTS) [219].

Neutron-based sensors rely on the collision between neutrons and hydrogen atoms present in the soil, which are mostly related to moisture content. After the collision, the neutrons are slowed down and can be detected and correlated with SWC. With a neutron thermalization probe, a radioactive source releases high-energy epithermal neutrons directly into the soil and then collects them for the final reading. A safer and more usable alternative in the field is cosmic ray neutron (CRN) sensing. This technology is based on the interaction between cosmic rays and the atmosphere of the Earth, producing high-energy neutrons that will eventually penetrate the soil and spread back into the atmosphere [148]. The now low-energy scattered neutrons are measured by CRN sensors near the soil surface. An example of a CRN sensor is the Finapp probe, developed by Finapp S.p.A. (Padova, Italy).

Electromagnetic sensors are currently the most widely used soil moisture sensing technology. They can measure the electrical properties of the soil and use them to calculate the final SWC. It is worth mentioning a currently used proximal SWC sensing technology based on electromagnetic fields: electromagnetic induction (EMI) technology [144,145]. EMI sensors operate by inducing an alternating current via the EM field generated from the transmitting coil and by measuring the resultant secondary field from the receiving coil [220]. Several differences between the two fields (amplitude, phase differences, and inter-coil spacing) are then used to calculate the apparent electrical conductivity ECa, which is related to SWC [220].

Other types of electromagnetic sensors are the so-called resistance-based sensors. These sensors measure SWC by creating a voltage difference between two electrodes and returning a resistance or EC value. SWC can then be calculated from the resulting resistance value, which decreases as the SWC increases. Some resistance-based commercial SWC sensors are the YL100 and YL69 soil moisture probes [138,150,151,152,153,154].

Electromagnetic sensors are also able to measure soil moisture from the charge-storing capacity of the soil via the measurement of the soil dielectric constant (εa) [141]. The measured soil capacitance has a nearly linear positive relationship with SWC due to the soil dielectric constant’s (εa) proportionality to soil moisture and independence of soil density, texture, salinity, and temperature [144,145]. These sensors are called dielectric (or permittivity) sensors, and they can be classified into time-domain reflectometry (TDR) sensors, time-domain transmission (TDT) sensors, resonant sensors, capacitance sensors (or frequency-domain reflectometry, FDR), and impedance-based sensors [138,141,144,145,155]. The HD3910.1/HD3910.12 probes (Senseca Germany Gmbh, Regenstauf, Germany) are an example of pure capacitive sensors. In TDR, an electric pulse is sent along the sensor rod with a certain velocity, and the time taken by the signal reflected along the rod to travel back is measured to determine the soil dielectric constant (or permittivity) εa and SWC [144,145,155]. Several sensors have been developed by adopting TDR technology, and they are now commercially available for measuring SWC along with other physicochemical properties, such as soil EC, soil pH, and soil temperature [122,150,155,157]: for example, the JXCT sensor (Weihai Jingxun Changtong Electronic Technology Co., Weihai, Shandong, China) [150,158] and the TDR 315/310 H sensors (Acclima Inc., Meridian, ID, USA) [155]. As with TDR, TDT measures the time an electrical pulse takes to travel over the length of the rod, but it is a closed circuit, and the time difference is measured from the connected beginning and end of the rods to the electrical source [144,155]. The TDT Aquaflex SI.99 soil sensor (Aquaflex soil moisture sensors, Onfarm Data, Middleton, Christchurch, New Zealand) is one of the commercially available TDT soil sensors [159]. Resonant sensors are based on the frequency/magnitude change within the sensor resonant circuit as the dielectric constant of the surrounding soil changes [147]. For capacitance sensors, the soil is used as a capacitor that stores part of an electric charge, and the measured frequency, used in precise equations to calculate SWC, depends on the capacitance [155]. FDR is often used as a synonym for capacitance, as they both use the soil as a capacitor and analyze the reflections of a continuous pulse, sometimes sent as a range of frequencies, although different probes are commercialized as either capacitance or FDR sensors [155]. Capacitance/FDR sensors are well implemented in the literature and commercialized [142,155,160,161,162,163,164,165]. For instance, the MEC10 sensor (Dalian Endeavor Technology Co., Dalian, China) [165] and the Drill and Drop sensor probe by Sentek (Sentek Sensor Technologies, Stepney, SA, Australia) use capacitance/FDR technology to measure soil properties [155]. The Drill and Drop probe (Sentek Sensor Technologies, Stepney, SA, Australia) is available from 30 to 120 cm in length, with sensors placed every 10 cm to measure soil temperature, moisture, and EC at different depths to obtain a complete view of the soil profile and state in the short or long term.

The impedance-based sensors take into consideration the dielectric constant and soil electrical conductivity, with a numerical solution of the Maxwell equation implemented in the microprocessor inside the probe to estimate the real and imaginary εa and SWC [155]. The single-depth Sensoterra sensor (Sensoterra BV, Enschede, Netherlands) is an impedance soil moisture sensor [157,178].

Ultrasonic-based soil sensors also measure the impedance difference between forward and backward pulses, although they implement an ultrasonic source and detector to measure acoustic impedance and send ultrasonic pulses [144,155]. The UGT582 soil moisture sensor (IFM electronic, Essen, Germany) is an example of an ultrasonic-based sensor [145].

### Appendix A.2

Several sensors have been developed to monitor soil temperature, such as thermocouples, resistance temperature detectors (RTDs), thermistors, diodes, and band-gap temperature (SBT) sensors [138,144,145].

Thermocouples use a pair of dissimilar (“different”) electrical conductors, forming a junction (namely the “hot” junction), and they are connected to a measuring device. When the hot junction is heated, a continuous flow of electrons begins in the circuit, producing a voltage that can be translated into temperature [144].

RTDs are temperature sensors made with a pure metal wire wrapped around a heat-resistant ceramic/glass core, while soil temperature is obtained by measuring the resulting voltage from a constant current bias [138,144,145]. One example of an RTD sensor is the Pt100 probe implemented by the TP32MTT.03.A/B sensors (Senseca Germany Gmbh, Germany), which can measure air and soil temperatures through a double-sensing probe.

Thermistors are temperature sensors based on electrical resistance and its dependence on temperature, similar to RTDs. However, thermistors are built from ceramic semiconductors or polymers, and they can be divided into two different groups: positive-temperature-coefficient thermistors or negative-temperature-coefficient thermistors [144]. Along with thermocouples, thermistors can be used in time-domain reflectometry (TDR)-based instruments to measure soil temperature, as the implementation of TDR technology for measuring soil temperature (namely, thermo-TDR) is expensive and has been little explored by sensor manufacturers until now. The ThetaProbe ML3 soil sensor (Delta-T Devices Ltd., Cambridge, UK) has a built-in thermistor for measuring soil temperature while also monitoring soil moisture and soil EC [151,155]. Moreover, the TDR 315 H and TDR 310 H soil sensors (Acclima Inc., Meridan, ID, USA) use thermistors for measuring soil temperature [155].

Diodes can be implemented in soil temperature monitoring due to the linear correlation between the junction temperature and junction voltage of the diode at low values of forward current: a change in temperature produces a change in voltage by a constant factor, called the K factor. An example of K-factor diodes is the SLT5006 soil sensor (Murata Manufacturing Co., Kyoto, Japan), which can also measure soil moisture and soil EC [171]. The SBT sensor technology is based on a temperature-dependent silicon diode forward voltage. The main advantage of an SBT sensor is that it can be integrated into a silicon integrated circuit (IC or “chip”) at a very low cost, with better monitoring performance [144]. The Belgian experimental Niphargus sensor and data logger [172] implements SBT technology with the ADT7410/ADT7420 probes (Analog Devices Inc., Wilmington, MA, USA).

### Appendix A.3

Soil pH can also be monitored through in situ soil sensing, although fewer available solutions have been found. Methods for measuring soil pH include colorimetric or photometric approaches (optical methods), conductometric or potentiometric approaches (electrochemical methods), and acoustic approaches [144,145].

The optical methods depend on pH-susceptible organic pigments and pH-color indicators to evaluate the pH values, and even though photometric strategies (i.e., optical fiber, fluorometric, holographic, and wide-range pH sensors) have greater resolution and reliability than colorimetric ones, neither of those techniques is appropriate for conducting in situ soil measurements [144,145].

Conductometric pH detectors contain conductivity electrodes and a pH-responsive material (hydrogel) applied as a thin film and used for detection, as a change in the external pH causes a reduction in the hydrogel-detecting layer, altering the electrical characteristics of the receiving tester [144,145]. Although traditional conductometric sensors are not suitable for in situ measurements, new technological advancements have been proposed to enable in situ soil pH conductometric monitoring [144,145].

The potentiometric soil pH sensor consists of two separate electrodes: one coated with pH-sensitive materials (glass or other materials) and the other made of inert materials to serve as the reference [144]. The pH value is obtained by comparing the potential difference between the two electrodes [144]. Examples of potentiometric sensors are solid dielectric pH probes with a large-area polytetrafluoroethylene (PTFE) liquid junction, which are made to last longer and be almost maintenance-free. The S-pH-**01-A** sensor (Seeed, Shenzhen, China) and the ZSPH soil pH sensor (ZATA IOT, Hong Kong, China) are examples of these sensors.

One example of acoustic-based soil pH sensors is the microcantilever-based pH sensor, in which the cantilever is fixed on top of a piezoresistive hydrogel or polymer layer that will shrink or swell according to changes in pH, leading to the deformation of the cantilever [144,145]. The cantilever-based pH sensor is highly sensitive to pH but more complex and more sensitive to noise [144,145].

Some alternatives for soil pH sensing include ion-selective field effect transistors (ISFETs) and magnetoelastic pH sensors [138,144,145,173]. An ISFET consists of three electrodes: a drain electrode, a source electrode, and a gate or reference electrode between the first two [138,144]. When an ISFET sensor is exposed to the testing material, the current from the source to the drain electrode will vary based on the pH change [138,144,173].

Magnetoelastic pH sensors, on the other hand, rely on an electromagnetic field that causes the substrate to vibrate, while the present pH-sensitive material starts to swell or shrink according to the pH [144,145]. The sensitive material volume change can affect the vibration frequency, and the pH change can be calculated from the interconversion between magnetic and elastic energy [144,145].

To reduce the costs of expensive probes, it is also possible to fabricate a custom printed soil pH sensor. Such a device can include an Alizarin (pH-sensitive redox compound 1,2-dihydroxyanthraquinone) composite working electrode coated with a Nafion membrane, a salt-membrane-coated reference electrode, and a simplified two-electrode architecture, allowing for more devices to capture continuous in-field information [175].

## Appendix B

The Food and Agriculture Organization of the United Nations (FAO) Harmonized World Soil Database (HWSD, v2.0) is a global soil database that offers detailed insights into soil morphology, chemistry, and physical characteristics, with 1 km resolution [221]. Launched in 2008 by the International Institute for Applied Systems Analysis (IIASA) and the Food and Agriculture Organization of the United Nations (FAO), the HWSD was a collaborative project involving other international organizations, such as the International Soil Reference and Information Center (ISRIC), the European Soil Bureau Network (ESBN), and the Institute of Soil Science at the Chinese Academy of Sciences (CAS) [221].

On the other hand, the United States Department of Agriculture (USDA)—Natural Resources Conservation Service (NRCS) Web Soil Survey (WSS) provides farmers, agencies, technical service providers, and others with access to relevant soil and related information needed to define land use and management in the United States [222]. Data are acquired through soil surveys and stored in a database. The use of digital soil maps and the Geographic Information System (GIS), coupled with soil attribute databases, enables the creation and elaboration of thematic soil maps [222].

The USDA National Agricultural Statistics Service Cropland Data Layer (NASS-CDL) is an annual geo-referenced, crop-specific land cover data grid produced using satellite imagery and agricultural ground reference data. In 2024, the NASS-CDL spatial resolution increased from 30 m to 10 m [223].

Google Earth Engine (GEE) is also used to download satellite images and data to analyze and train predictive models [60]. GEE combines a multi-petabyte catalog of satellite imagery with planetary-scale analysis capabilities, including more than thirty years of daily updated scientific datasets [224].
