# Supplementary figures and images for: A Systematic Review of Soil Properties to Support Mycotoxin Model Development with In-Field Soil Sensing
Source: Sensors (Basel). 2026 Jun 25;26(13):4044. doi: 10.3390/s26134044 (PMC13364116; doi:10.3390/s26134044)

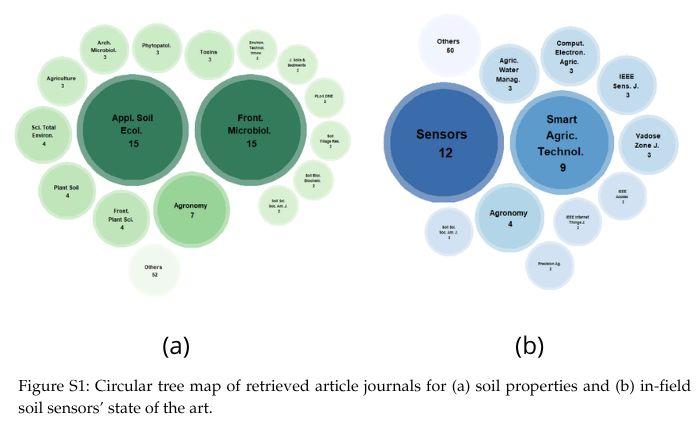

Supplement: Supplementary file 1 [file sensors-26-04044-s001.zip › Figure S1.png]

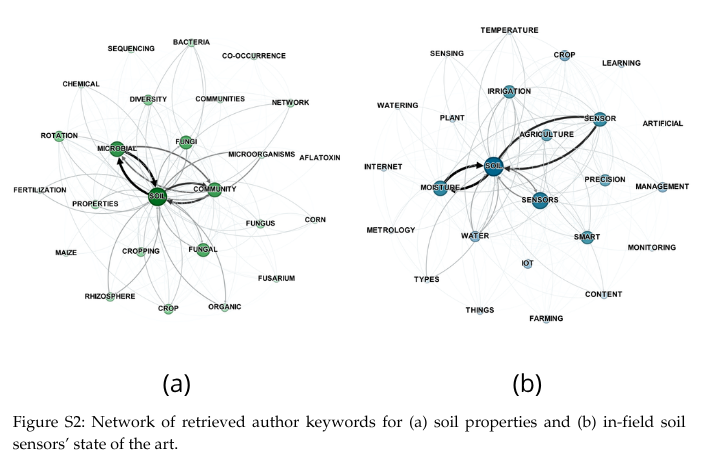

Supplement: Supplementary file 1 [file sensors-26-04044-s001.zip › Figure S2.png]

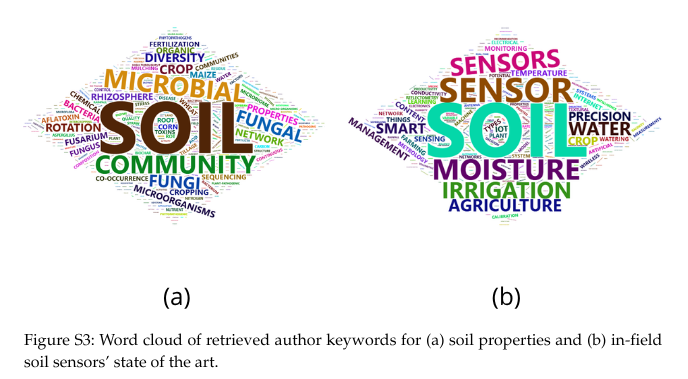

Supplement: Supplementary file 1 [file sensors-26-04044-s001.zip › Figure S3.png]
